# Cutaneous leishmaniasis: emerging insights in epidemiology, diagnosis, and treatment

**DOI:** 10.3389/fphar.2026.1744348

**Published:** 2026-02-17

**Authors:** Navin Kumar, Dilip K. Kakru, Rekha Arcot, Sorabh Lakhanpal, Sujeet K. Singh, Sanjay Kumar, Jeffrin Reneus Paul

**Affiliations:** 1 Department of Biotechnology, Graphic Era Deemed University, Dehradun, India; 2 Centre for Promotion of Research, Graphic Era Hill University, Dehradun, India; 3 Department of Microbiology, Sharda University, Greater Noida, India; 4 Department of Surgery, Dr. D. Y. Patil Medical College, Hospital and Research Centre, Dr. D. Y. Patil Vidyapeeth (Deemed-to-be-University), Pimpri/Pune, Maharashtra, India; 5 School of Pharmaceutical Sciences, Lovely Professional University, Phagwara, India; 6 Department of Biotechnology, Noida Institute of Engineering and Technology (Pharmacy Institute), Greater Noida, India; 7 Centre for Research Impact and Outcome, Chitkara College of Pharmacy, Chitkara University, Rajpura, Punjab, India; 8 Saveetha Medical College and Hospital, Saveetha Institute of Medical and Technical Sciences, Saveetha University, Chennai, India

**Keywords:** cutaneous leishmaniasis, diagnosis, neglected tropical diseases, sandflies, treatment

## Introduction

Cutaneous leishmaniasis (CL) is a parasitic disease caused by protozoa of the genus Leishmania, transmitted by infected female sandflies (Phlebotomus and Lutzomyia species). The World Health Organization (WHO) classifies CL among the neglected tropical diseases, currently estimates 600,000 to 1 million new cases occur worldwide annually (WHO, Leishmaniasis Fact sheets, 2023). The expanding distribution of CL, atypical species–disease associations, and persistent care gaps emphasize the need for updated evidence synthesis. This review synthesizes recent epidemiological, diagnostic, and therapeutic developments across endemic and emerging regions and offers an integrated framework to guide surveillance strategies, clinical decision-making, and future research.

## Epidemiology and risk factors

Globally, CL displays marked geographical heterogeneity. The WHO identifies high endemicity in the Middle East, Central Asia, North Africa, and Latin America (de Vries and Schallig, 2022). Environmental change, population displacement, and urbanization have been associated with rising incidence in several regions. In Colombia, a large-scale ecological study of 121,828 cases from 2007 to 2021 identified substantial spatial variability, with standardized incidence rates between 0 and 16,072 per 100,000 population (Tapias Rivera et al., 2025). Factors such as migration, forest coverage, and poverty correlated with higher CL risk, while rainfall and urbanization appeared protective. In India, CL is geographically focal and historically concentrated in the hot, arid north-western belt, especially Rajasthan (Bikaner/Thar Desert region). In recent years, India has also reported emerging or non-traditional foci, notably in Himachal Pradesh (Lypaczewski et al., 2024; Aara et al., 2013). In Diyala Province, Iraq, a 10-year retrospective analysis of 25,474 confirmed cases showed a high burden among children aged 5–14 years (33%), with seasonality peaking in winter months (November–February) (Hamad et al., 2025). Similarly, in Pakistan reported increasing endemicity in non-traditional areas of Punjab with Leishmania tropica identified as the dominant species (Ashraf et al., 2025).

Across the studies summarized in Table 1, consistent epidemiological patterns emerge, including a higher burden among children and young adults, frequent male predominance, and marked seasonality aligned with sandfly activity, typically peaking in warmer or post-rainy periods. Transmission is often peri-domestic or rural but increasing urban and intradomiciliary exposure has been documented in several settings, reflecting changing vector behaviour. In India and South Asia, these global patterns intersect with arid or ecologically suitable environments, socioeconomic vulnerability, and emerging non-traditional foci, reinforcing the contribution of CL to the regional and global disease burden. Sociodemographic factors are key determinants of CL risk and awareness. In a cross-sectional study from Quetta, Pakistan, 63.9% of individuals had experienced CL, but knowledge of preventive measures was limited (19%), particularly among women, individuals with lower education, and rural residents (Ali et al., 2025).

**TABLE 1:** Epidemiology and risk factors associated with CL across endemic regions.

Country/Region	Study (year)	Study design & population	Dominant *Leishmania* species	Key epidemiological findings	Identified risk factors
Pakistan (Balochistan, Hazara Community)	Ali et al. (2025)	Cross-sectional (N = 216)	*L. tropica* (presumed)	63.9% had CL; awareness high but preventive knowledge low (19%)	Female gender, low education, rural residence, poor housing, low socioeconomic status
Pakistan (Punjab, Khushab District)	Ashraf et al. (2025)	Cross-sectional molecular profiling (N = 423)	*L. tropica* (confirmed by ITS1-RFLP)	41.8% microscopy positive; peak in January (19.3%)	Female gender (57%), age <20 years, single facial lesions, low rainfall, 22 °C–27 °C temperature
Pakistan (Khyber Pakhtunkhwa)	Uddin et al. (2025)	Retrospective survey (2022–2023, N = 2035)	*L. tropica*/*L. major*	High prevalence (61.9%) in <20 years; 52.5% males	Outdoor activity, facial exposure, warm season (spring/summer)
Iraq (Diyala Province)	Hamad et al. (2025)	Retrospective (2011–2021, 25,474 cases)	*L. major* (likely)	Annual peaks: 4,425 cases in 2015; winter seasonality	Children aged 5–14 years, female gender (52%), poor housing, rural exposure
Iran (Damghan County)	Pourmohammadi et al. (2025)	Ecological time-series (2012–2021)	*L. major*	Incidence correlated with climate factors	Relative humidity, sunshine hours, air pressure, high temp (P < 0.05)
Colombia (Nationwide)	Tapias Rivera et al. (2025)	Bayesian ecological model (2007–2021, 121,828 cases)	*L. panamensis*, *L. braziliensis*	Median annual cases 7,605; incidence 0–16,072 per 100,000	Poverty, forest coverage, internal migration ↑; rainfall ↓ CL incidence
Algeria (Sahara Desert, Djamaa Province)	Boulal et al. (2025)	12-year retrospective analysis (2012–2023, 4,436 cases)	*L. major*	Mean annual incidence: 369.7; seasonality: peak in Nov & Jan	Male sex (65.2%), teenagers (10–20 years), outdoor exposure, lower limbs
Brazil (Montezuma, Minas Gerais)	Lopes et al. (2025)	Entomological survey	*L. braziliensis* (vector)	Intra-domiciliary sandfly vector presence confirmed	Indoor presence of *Nyssomyia intermedia*, domestic transmission risk
Sri Lanka (Anuradhapura District)	Gunasekara et al. (2025)	Qualitative, community-based	*L. donovani* (cutaneous strain)	Significant psychosocial & financial burden	Delayed diagnosis, poor access to healthcare, stigma
Iran (Isfahan Province, Military Personnel)	Saneian et al. (2025)	Interventional (ATSB trial, 2012–2022)	*L. major*	Decline in cases post-intervention (196 → 55/year)	Climate, sandfly density, vector exposure; reduction not statistically significant
Portugal (Imported Case)	de Carvalho et al. (2025)	Case report	*L. mexicana*	First imported CL case in Portugal	Travel history, imported infection risk
Uzbekistan (Pediatric HIV Case)	Dadaboev et al. (2025)	Case report	*L. major*	Diffuse CL as first sign of HIV infection	Immunosuppression, misdiagnosis (scabies, Kaposi’s sarcoma)
India (Rajasthan)	Aara et al. (2013)	Large case series	*L. tropica*	Male predominance and association with lower socioeconomic groups	Peri-domestic exposure, arid climate ecology
India (Himachal Pradesh)	Lypaczewski et al. (2024)	Case report	*L. donovani*	changing atypical species–disease patterns	Emergence linked to parasite diversification and ecological/vector shifts
Bangladesh	Khan et al. (2019)	Case report	*L. major*	Imported CL case	Travel/migration exposure

## Climatic and environmental influences

Environmental changes, including deforestation and broader climate change, have been linked to rising transmission rates of CL in multiple regions. Deforestation alters reservoir and vector habitats, promoting closer contact between humans, sandflies, and animal hosts. For example, in the Amazon basin and parts of South America (Brazil, Colombia, Peru), rapid forest clearance for agriculture and infrastructure has been associated with increased CL incidence, as sandfly vectors and sylvatic reservoir hosts expand into disturbed landscapes (Olivera et al., 2025; De Oliveira et al., 2021). Similarly, climate warming trends have expanded the altitude and latitude of sandfly survival, contributing to the emergence of CL in previously non-endemic highland regions of Andean countries and southern Brazil. These ecological shifts illustrate how anthropogenic environmental change can disrupt endemic stability, alter vector ecology, and increase human disease risk across continents. In north-west Pakistan, Uddin et al. reported that CL incidence peaked during summer and spring, corresponding to optimal sandfly breeding conditions (Uddin et al., 2025). These observations align with seasonal patterns documented in the Sahara Desert of Algeria, where cases peaked in November and January, reflecting vector transmission cycles (Boulal et al., 2025).

## Vector and reservoir ecology

Vector ecology is central to understanding CL transmission. CL is caused by various *Leishmania* species. In Europe, Asia & Africa, *L. major*, *L. tropica*, *L. aethiopica, L. infantum*, and *L. donovani* are common, while in United States of America, *L. mexicana* and *L. braziliensis* predominate (Table 1). CL caused by *L. donovani & L. infantum* is an atypical manifestation of a parasite traditionally associated with visceral leishmaniasis. *L. donovani* MON-37 classically a visceralizing parasite elsewhere has emerged as a major cause of CL in some settings, highlights that visceral lineages can become established as cutaneous pathogens under certain evolutionary and ecological pressures. In certain endemic regions, particularly Sri Lanka and parts of East Africa, *L. donovani* causes localized cutaneous lesions instead of systemic disease (Gunasekara et al., 2025). *L. infantum* is mainly found in the Mediterranean region, the Middle East, and North Africa, where transmission is zoonotic, with dogs as the primary reservoir. Transmission occurs through infected sandflies, with rodents, hyraxes, and other mammals serving as natural reservoirs (Pareyn et al., 2025). CL is transmitted by female sand flies in the genera Phlebotomus (Europe, Asia & Africa) and Lutzomyia (United States of America) (Gunasekara et al., 2025). Host reservoirs of CL are primarily mammals that maintain *Leishmania* parasites in nature and facilitate transmission to humans through sandfly bites. Major reservoirs include rodents such as gerbils (*Rhombomys opimus*) and jirds (*Meriones* spp.) for *L. major* in the Old World, and hyraxes for *L. aethiopica* in East Africa. In the New World, forest rodents, opossums, and sloths serve as reservoirs for *L. mexicana* and *L. braziliensis* complexes (Pareyn et al., 2025; Saliba and Oumeish, 1999). Humans may act as reservoirs in *anthroponotic* forms like *L. tropica* (Saliba and Oumeish, 1999). Reservoir ecology depends on species, geography, and environment, influencing disease persistence and transmission patterns. After ingesting amastigotes from an infected host, parasites develop as promastigotes in the fly gut and are inoculated at the next blood meal; vector competence is species-specific. In Brazil, *Nyssomyia intermedia* was identified as a potential intradomiciliary vector in Montezuma, capturing 96.7% of sandflies within residential areas (Lopes et al., 2025). This highlights a growing trend toward domestic transmission in regions traditionally associated with sylvatic cycles. Integrated entomological surveillance and housing improvements are therefore essential for prevention.

## Pathogenesis and immunological insights

CL occurs when infected sandflies inoculate Leishmania promastigotes into the skin, where they are phagocytosed by macrophages and differentiate into amastigotes. Through immune evasion strategies, including modulation of phagolysosomal function and cytokine responses, parasites persist. A Th1-dominant response is associated with parasite clearance and healing, whereas Th2-skewed immunity promotes chronic disease and persistent ulcerative lesions (Scott and Novais, 2016). Host immunity plays a key role in disease outcome. Gashaw et al. demonstrated significantly lower CD4^+^ T-cell counts among Ethiopian CL patients compared with controls, suggesting immunosuppression as a factor in disease severity (Gashaw et al., 2025). Genetic polymorphisms in cytokine genes (IL10, IL4, IFNG, TNFA), HLA class II loci, and NRAMP1 (SLC11A1) influence immune regulation, antigen presentation, and macrophage microbicidal activity, thereby affecting susceptibility, lesion severity, and clinical outcome in CL (Mohamed et al., 2003; Blackwell et al., 2009). Together, these genetic differences modulate immune balance and determine disease progression and healing outcomes.

## Symptoms & diagnostic advances

CL typically begins as a painless papule that gradually enlarges into a nodule and may ulcerate, forming a well-demarcated lesion with raised margins and a central crust. CL caused by *L. infantum* has been increasingly reported in the Americas, where this species traditionally associated with visceral disease can present as strictly cutaneous infection in immunocompetent and immunocompromised individuals.

Beyond conventional microscopy and culture, newer diagnostic approaches are under active investigation. Artificial intelligence based microscopy systems, such as the YOLOv8 model described by Gadri et al., demonstrated high diagnostic accuracy in a laboratory-based validation study, but their use in field settings remains limited by infrastructure and equipment requirements (Gadri et al., 2025). Molecular diagnostics, particularly PCR performed on non-invasive cutaneous swabs, have shown high sensitivity in case-based studies and small clinical series, including among immunocompromised patients (Povolo et al., 2025). Rapid antigen detection tests and isothermal DNA amplification techniques such as loop-mediated isothermal amplification (LAMP) or recombinase polymerase amplification (RPA) have demonstrated promising sensitivity and specificity in pilot studies for CL. These assays enable field-applicable detection of Leishmania DNA without the need for thermocyclers.

## Therapeutic developments

### Clinically established therapies

Pentavalent antimonials continue to be widely used but are limited by toxicity and emerging resistance. Liposomal amphotericin B has an established clinical role, particularly in older patients and those with contraindications to antimonials. Clinical trial data indicate that cumulative doses of 24 mg/kg achieve high cure rates with acceptable safety profiles (Azouz et al., 2025).

### Preclinical and experimental approaches

Several novel therapies are still at a preclinical stage, with amphotericin B–retinoic acid liposomal formulations demonstrating promising immunomodulatory and anti-lesional effects in animal models but lacking clinical evaluation (Santos et al., 2025). Topical microemulsions with *Libidibia ferrea* phenolics and photoactivated hypericin nanoparticles show experimental efficacy, but additional pharmacokinetic, safety, and clinical studies are needed prior to clinical application (Jensen et al., 2025). De Oliveira et al. demonstrated that photoactivated hypericin nanoparticles induced apoptosis *in L*. *amazonensis* by inhibiting trypanothione reductase, offering a promising nanomedicine approach (de Oliveira et al., 2025).

### Prevention and control

Vector control and environmental management are the cornerstone of CL prevention but are increasingly challenged by insecticide resistance, sandfly adaptation, urbanization, and climate variability. While Attractive Toxic Sugar Baits have shown promise, their scalability and long-term impact remain unclear (Saneian et al., 2025). Further challenges include weak surveillance, underreporting, limited incorporation of CL into national programs, and low community awareness. Climatic forecasting models offer early-warning potential, but their impact relies on consistent data availability and public-health action (Pourmohammadi et al., 2025; Majidnia et al., 2023). Effective prevention will require integrated vector management, improved housing conditions, community engagement, and region-specific strategies.

## Conclusion

CL remains a significant and evolving public-health challenge, shaped by ecological change, socioeconomic vulnerability, and parasite diversity. Although advances in diagnostics, therapeutics, and predictive modeling have expanded the available tools for control, their impact is constrained by health-system limitations and inequitable access. The emergence of atypical disease patterns, particularly in South Asia, underscores the need for strengthened surveillance and species-specific approaches. Future progress will depend on multidisciplinary strategies that integrate molecular epidemiology, vector ecology, patient-centered care, and sustainable prevention programs to reduce the global burden of CL.

## References

[B1] Aara N. Khandelwal K. Bumb R. A. Mehta R. D. Ghiya B. C. Jakhar R. (2013). Clinco-epidemiologic study of cutaneous leishmaniasis in Bikaner, Rajasthan, India. Am. J. Trop. Med. Hyg. 89 (1), 111–115. 10.4269/ajtmh.12-0558 23716414 PMC3748465

[B2] Ali Z. Khan M. H. Akbar A. Bahar T. Ali H. Shoaib F. (2025). The sociodemographic risk factors associated with cutaneous leishmaniasis of the hazara community of quetta, Balochistan, Pakistan: a cross-sectional study. Health Sci. Rep. 8 (10), e71268. 10.1002/hsr2.71268 41080033 PMC12511313

[B3] Ashraf A. Qadeer S. Bukhari U. A. Zeb I. Rasool A. Salma U. (2025). Molecular profiling and risk factors assessment of cutaneous leishmaniasis in an expanding endemic focus of Punjab, Pakistan. Parasitol. Res. 124 (11), 126. 10.1007/s00436-025-08581-2 41205103 PMC12596330

[B4] Azouz S. F. Lago E. L. Guimaraes L. H. Nolasco S. Suprien C. Carvalho E. M. (2025). Treatment of cutaneous leishmaniasis with liposomal Amphotericin B in the elderly: a randomized clinical trial. Am. J. Trop. Med. Hyg. 10.4269/ajtmh.24-0812 41187344 PMC12781429

[B5] Blackwell J. M. Fakiola M. Ibrahim M. E. Jamieson S. E. Jeronimo S. B. Miller E. N. (2009). Genetics and visceral leishmaniasis: of mice and man. Parasite Immunol. 31 (5), 254–266. 10.1111/j.1365-3024.2009.01102.x 19388946 PMC3160815

[B6] Boulal B. Bendjoudi D. Leulmi H. Chenchouni H. (2025). A Bayesian modeling and retrospective analysis of cutaneous leishmaniasis in the Sahara Desert. Acta Trop. 271, 107893. 10.1016/j.actatropica.2025.107893 41173066

[B7] Dadaboev A. Solmetova M. Shukurova M. Kotamjani S. S. (2025). Diffuse cutaneous leishmaniasis caused by Leishmania major as the initial presentation of HIV misdiagnosed as scabies and Kaposi's sarcoma: a case report. Iran. J. Parasitol. 20 (3), 482–488. 10.18502/ijpa.v20i3.19626 41181207 PMC12579477

[B8] de Carvalho M. M. Cortes S. Cristovao J. M. Cardoso J. C. Catorze M. G. Miroux-Catarino A. (2025). Cutaneous leishmaniasis due to Leishmania (L.) mexicana: first imported case reported in Portugal. An Bras Dermatol 100 (6), 501223. 10.1016/j.abd.2025.501223 41151368 PMC12596591

[B9] De Oliveira R. A. C. Miranda C. Guedes J. A. Filgueiras T. G. M. Bichara C. N. C. Araujo M. S. (2021). Cutaneous leishmaniasis in protected environmental areas in the Eastern Amazon: the case of Sao Felix do Xingu, Para, Brazil. J. Infect. Dev. Ctries. 15 (11), 1724–1730. 10.3855/jidc.13936 34898502

[B10] de Oliveira L. F. dos Reis A. B. Bueno A. P. R. Suzawa T. Gonçalves M. J. S. Junior A. S. (2025). Photoactivity of Pluronic(R) F-127 nanoencapsulated hypericin against Leishmania: a promising approach for cutaneous leishmaniasis. J. Photochem Photobiol. B 273, 113292. 10.1016/j.jphotobiol.2025.113292 41176882

[B11] de Vries H. J. C. Schallig H. D. (2022). Cutaneous leishmaniasis: a 2022 updated narrative review into diagnosis and management developments. Am. J. Clin. Dermatol 23 (6), 823–840. 10.1007/s40257-022-00726-8 36103050 PMC9472198

[B12] Gadri S. Bounab S. Benazi N. Zerouak F. (2025). A new diagnostic method and tool for cutaneous leishmaniasis based on artificial intelligence techniques. Comput. Biol. Med. 192 (Pt B), 110313. 10.1016/j.compbiomed.2025.110313 40359677

[B13] Gashaw B. Yizengaw E. Nibret E. (2025). Low CD4+ T-cell count and haematological parameters in patients with cutaneous leishmaniasis, Northwest Ethiopia: a cross-sectional study. Trans. R. Soc. Trop. Med. Hyg. 10.1093/trstmh/traf120 41143869

[B14] Gunasekara S. D. Wickramasinghe N. D. Weerakoon K. G. Fernando M. S. Shanthapriya S. H. Price H. P. (2025). Understanding the complexity of cutaneous leishmaniasis patient journey in endemic rural Sri Lanka: a qualitative study. BMC Infect. Dis. 25 (1), 1448. 10.1186/s12879-025-11962-8 41168716 PMC12574255

[B15] Hamad E. M. Ghaffarifar F. Dalimi A. (2025). Cutaneous Leishmaniasis in Diyala Province from Eastern part of Iraq during the period of 2011 to 2021. Iran. J. Parasitol. 20 (3), 400–407. 10.18502/ijpa.v20i3.19615 41181198 PMC12579472

[B16] Jensen B. B. Cardoso Rodrigues Dos Santos K. S. Comandolli-Wyrepkowski C. D. Araujo da Silva F. M. Coutinho de Souza C. da Paz Lima M. (2025). Topical microemulsion with phenolic compounds from Libidibia ferrea (Fabaceae): an alternative treatment for cutaneous leishmaniasis. Eur. J. Microbiol. Immunol. (Bp). 10.1556/1886.2025.00064 41196291 PMC12771332

[B17] Khan M. A. A. Chowdhury R. Nath R. Hansen S. Nath P. Maruf S. (2019). Imported cutaneous leishmaniasis: molecular investigation unveils Leishmania major in Bangladesh. Parasit. Vectors 12 (1), 527. 10.1186/s13071-019-3771-6 31699125 PMC6836376

[B18] Lopes B. T. Oliveira C. H. Souza P. A. A. Cordeiro-de-Barros R. M. Teixeira T. J. Santos R. M. D. (2025). Ocurrence of Nyssomyia intermedia (Diptera: psychodidae) in intradomiciliary environments: a potential vector of american cutaneous leishmaniasis in Montezuma, 2024. Epidemiol. Serv. Saude. 34, e20250198. 10.1590/S2237-96222025v34e20250198.en 41172443 PMC12560226

[B19] Lypaczewski P. Chauhan Y. Paulini K. Thakur L. Chauhan S. Roy E. I. (2024). Emerging Leishmania donovani lineages associated with cutaneous leishmaniasis, Himachal Pradesh, India, 2023. Emerg. Infect. Dis. 30 (9), 1957–1959. 10.3201/eid3009.231595 39174021 PMC11346985

[B20] Majidnia M. Ahmadabadi Z. Zolfaghari P. Khosravi A. (2023). Time series analysis of cutaneous leishmaniasis incidence in Shahroud based on ARIMA model. BMC Public Health 23 (1), 1190. 10.1186/s12889-023-16121-9 37340451 PMC10283195

[B21] Mohamed H. S. Ibrahim M. E. Miller E. N. Peacock C. S. Khalil E. A. Cordell H. J. (2003). Genetic susceptibility to visceral leishmaniasis in the Sudan: linkage and association with IL4 and IFNGR1. Genes Immun. 4 (5), 351–355. 10.1038/sj.gene.6363977 12847550

[B22] Olivera M. J. Porras-Villamil J. F. Fuentes M. V. (2025). Outbreaks and incidence of vector-borne diseases in Colombia (2007-2024): impact of climate change and deforestation. Biomedica 45 (Sp. 2), 17–29. 10.7705/biomedica.7897 41325564 PMC12752917

[B23] Pareyn M. Alves F. Burza S. Chakravarty J. Alvar J. Diro E. (2025). Leishmaniasis. Nat. Rev. Dis. Prim. 11 (1), 81. 10.1038/s41572-025-00663-w 41266459

[B24] Pourmohammadi B. Shahsavan F. Paknazar F. Manavi M. Fatemi F. (2025). Climatic factors and their impact on cutaneous Leishmaniasis: predicting the number of patients using ARIMA model. Parasitol. Int. 111, 103185. 10.1016/j.parint.2025.103185 41175924

[B25] Povolo L. Barbiero A. Spinicci M. Petrosillo N. Bartoloni A. Zammarchi L. (2025). Cutaneous leishmaniasis in the immunocompromised: diagnostic and therapeutic insights from a case documented in central Italy. Infect. Dis. Rep. 17 (5), 125. 10.3390/idr17050125 41148639 PMC12564556

[B26] Saliba E. Oumeish O. Y. (1999). Reservoir hosts of cutaneous leishmaniasis. Clin. Dermatology 17 (3), 275–277. 10.1016/s0738-081x(99)00045-0 10384866

[B27] Saneian M. Barati M. Hosseini-Shokouh S. J. Dabbagh-Moghaddam A. Mohebali M. Mirabedini Z. (2025). Effectiveness of attractive toxic sugar baits (ATSB) in reducing cutaneous leishmaniasis among military personnel in Isfahan province, central part of Iran. Iran. J. Parasitol. 20 (3), 337–345. 10.18502/ijpa.v20i3.19608 41181202 PMC12579439

[B28] Santos T. T. Lages E. B. Ricotta T. N. Q. de Oliveira L. G. Ramos G. S. Burlot J. (2025). Long-circulating liposomes codelivering amphotericin B and retinoic acid for cutaneous leishmaniasis treatment. ACS Omega 10 (41), 48514–48530. 10.1021/acsomega.5c06156 41141727 PMC12547526

[B29] Scott P. Novais F. O. (2016). Cutaneous leishmaniasis: immune responses in protection and pathogenesis. Nat. Rev. Immunol. 16 (9), 581–592. 10.1038/nri.2016.72 27424773

[B30] Tapias Rivera J. Martinez-Vega R. Quintero-Garcia W. L. Torres-Martinez D. S. Monroy-Diaz A. L. Sanchez-Corrales L. (2025). Climatic, socioeconomic, and migratory factors on the epidemiological dynamics of cutaneous leishmaniasis in Colombia, 2007-2021. PLoS Negl. Trop. Dis. 19 (10), e0013594. 10.1371/journal.pntd.0013594 41082517 PMC12517516

[B31] Uddin S. Khan I. Rehman A. U. Rahman H. U. Hizbullah (2025). Hizbullah. Epidemiological patterns of cutaneous leishmaniasis in north-west Pakistan (2022-2023). BMC Public Health 25 (1), 3480. 10.1186/s12889-025-23175-4 41088089 PMC12522337

